# OMI-based emission source classification in East China and its spatial redistribution in view of pollution control measures

**DOI:** 10.1007/s10661-024-12421-8

**Published:** 2024-02-29

**Authors:** Marina Zara, Ronald van der A, Jieying Ding, Trissevgeni Stavrakou, Folkert Boersma

**Affiliations:** 1 Wageningen University and Research (WUR), Wageningen, Netherlands; 2 Now at Karlsruhe Institute of Technology (KIT), Karlsruhe, Germany; 3 Royal Netherlands Meteorological Institute (KNMI), De Bilt, Netherlands; 4 Royal Belgian Institute for Space Aeronomy (BIRA-IASB), Brussels, Belgium

**Keywords:** Nitrogen oxides, Sulphur dioxide, China, OMI, Emission sources, Air pollution control measures

## Abstract

This study aims to generate a satellite-based qualitative emission source characterization for the heavily polluted eastern part of China in the 2010–2016 time period. The applied source identification technique relies on satellite-based NO_x_ and SO_2_ emission estimates by OMI, their SO_2_:NO_x_ ratio, and the MIX anthropogenic emission inventory to distinguish emissions from different emission categories (urban, industrial, natural) and characterize the dominant source per 0.25° × 0.25° grid cell in East China. Overall, we find good agreement between the satellite- and emission inventory–based spatiotemporal distribution and characterization of the dominant emission sources in East China in 2010–2016. In 2010, the satellite measurements suggest an emission distribution less dominated by industrial areas, a somewhat larger role for urban/transportation areas and agricultural activities, and more natural emissions in the southern part compared to the bottom-up emission categorization. In 2016, more than half of the classified emission categories over East China have remained the same. At the same time, there is a notable increase of agricultural lands and decrease of areas dominated by industry/transportation in 2016, suggestive of an overall decrease in heavy air pollution in East China over the course of 7 years. This is likely attributed to the sustained efforts of the Chinese government to drastically improve the air quality, especially since 2013 when the National Air Pollution Prevention and Control Action Plan was enacted. However, signs of urban expansion (urbanization) and rural–urban migration (“Go West” motion) stemmed from China’s rapid economic growth and labour demand are evident; escalating industrialization (even with cleaner means) and the urban population growth in East China resulted in stronger emissions from sources representing consumption and transportation which are strongly related to NO_2_ and PM_10_ pollution (rather than SO_2_) and are directly influenced by the population size. This resulted to a shift of the emissions from the east mainly to the north and northwest of East China. Overall, although the effectiveness of the Chinese environmental control policies has been successful, the air pollution problem remains an important concern.

**Supplementary Information:**

The online version contains supplementary material available at 10.1007/s10661-024-12421-8.

## Introduction

Air pollution has been a major concern since the 1980s in East China, and extremely important in the last decade. It is a major cause for premature death, respiratory problems, diseases (World Health Organization, WHO, Air Quality and Health, https://www.who.int), and environmental damage (EEA Technical report, 2014; Manisalidis et al., 2020). Systematic exposure to high levels of sulphur dioxide (SO_2_) induces headaches, anxiety, and cardiovascular diseases, while nitrogen oxides (NO_x_ = NO + NO_2_) are responsible for irritation of eyes and breathing problems, inflammation, infections, asthma and reduced lung function, impacts on liver, spleen, and blood (WHO; EEA 2020). High NO_x_ levels disrupt terrestrial and aquatic ecosystems through deposition of excessive amounts of nitrogen nutrients leading to eutrophication. SO_2_ also contributes to the acidification of soil, lakes, and rivers, causing loss of biodiversity (EEA 2020).

NO_x_ and SO_2_ emissions in China are often located within the premises of highly populated cities with intense industrialization and transit veins: strong coal- and sulfur-based fuel combustion lead to dangerously high NO_x_ and SO_2_ emissions that directly impact the local air quality. This study assesses the spatiotemporal distribution and magnitude of NO_x_ and SO_2_ emissions, and their SO_2_:NO_x_ ratio, which is indicative of changes in the fuel combusted and can be used as a proxy for downwind secondary PM_2.5_ aerosols (particulate matter with an aerodynamic diameter less than 2.5 μm) formation (e.g., Chen et al., 2021; Guerra et al., 2014). Previous studies have shown that the sulphate–nitrate–ammonium (SNA) aerosols account for 20–57% of PM_2.5_ in China (Wang et al., 2011; Huang et al., 2014; Liu et al., 2018; Chen et al., 2021). Therefore, changes in reactive NO_x_ and SO_2_ emissions can strongly affect the SNA fraction of PM_2.5_ and consequently worsen the local air quality.

The severity of air pollution prompted action by the Chinese authorities (e.g., Ouyang, 2013; Huang et al., 2017). National large-scale control policies have been initiated and implemented with the goal to reduce SO_2_ emissions from coal-burning in industry and power plants, and NO_x_ emissions also from vehicles (e.g. Jin et al., 2016; van der A et al., 2017; Xu et al., 2021). After a period of strong economic activity with few limits to emissions, measures have been implemented since 2012 including desulfurization, denitrification, and dust precipitation of power plants and big industry boilers, closing down small coal mines, phase-out of outdated industrial capacity, use of low-sulfur coal, and suspension of clinker production during the heating season (in most areas of the North) (Xu et al., 2021). As vehicles are important contributors to NO_x_, the early retreat of old vehicles and higher fuel emission standards for gasoline and diesel cars have been part of the National Air Pollution Prevention and Control Action Plan (2013). A shift to cleaner forms of energy (coal to gas) both for industry and transportation complemented the air pollution control policies while raising public awareness to the importance of clean air (Jin et al., 2016; van der A et al., 2017; Xu et al., 2021). Zhao et al. (2018) report an astounding decrease of ~ 75% of SO_2_ concentration in 2015 relative to 1996 in contrast to a ~ 23% increase of NO_x_ for the same time period, rendering NO_x_ emissions the main air pollution reduction target: by 2015, 25% of the world’s total emitted NO_x_ load comprised China’s NO_x_ emissions alone (Cui et al., 2013). The policy of drastically reducing NO_x_ emissions is potentially contradicted by China’s 2014–2020 urbanization, a strategic plan for economic development and enhancement of interregional equality and national security. Chu (2020) reported that the plan led to the effective migration of rural populations into urban regions, relocating secondary industries to inland regions, and unintentionally provided excuses for land expropriation (also supported by statistics on land-sale revenue). Although some secondary industries have been relocated from eastern coastal cities to inland locations and the nodal roles of some inland cities have been enhanced, population movements have not been channelled to city clusters in the inland regions or to smaller cities. China has witnessed fast urbanization in past decades, with the urbanization rate increasing from 11.2% in 1950 to 59.6% in 2018, with the population migration from rural to urban regions acting as the major driving force (Shi et al., 2022). In addition, the population doubled by 2018 in the span of less than 20 years, among which more than 50% are migrants from rural to urban regions. The objective of optimizing the patterns of urban expansion has been achieved only partially. Such operations are anticipated to impact the emissions strength and redistribute emission sources across the land.

The distribution and type of sources that emit air pollutants are investigated and documented in emission inventories. Emission inventories (i) list the types of (point, line, area) sources for a certain region, (ii) determine the list of pollutants emitted from said sources, (iii) determine the emission factor related to each pollutant, and finally (iv) determine the magnitude of each source. With coherent information from in situ measurements and governmental archives, a ground-based emission estimate is obtained for each emission source and pollutant. Here, we turn to independent satellite measurements of air pollution to classify SO_2_ and NO_x_ emission sources in East China and compare our classification to commonly used emission inventories. Our motivation is to provide a qualitative assessment of the dominant emission sources in East China from the satellite perspective, complementary to the ground-based emission inventories, particularly when information from emission inventories is unavailable (e.g., due to spatiotemporal gaps in field measurements) or outdated (e.g., due to obsolete emission factors). This approach concerns nitrogen dioxide (NO_2_) and SO_2_ tropospheric column measurements observed by the space-borne Ozone Monitoring Instrument (OMI) from which we infer NO_x_ and SO_2_ emission estimates via inverse modeling with the DECSO (Daily Emission estimates Constrained by Satellite Observations) algorithm (Ding et al., 2017). We use these top-down NO_x_ and SO_2_ emission estimates, and their SO_2_:NO_x_ ratio, to classify dominant emission sources and also monitor spatiotemporal changes in their distribution following the implementation of environmental policies in the period 2010–2016, a period that denotes a strong decrease of SO_2_ emissions and a rise followed by a moderate fall of NO_x_ emissions. The main focus of this work is the strongly related to combustion processes SO_2_ and NO_x_ emissions rather than the volatile organic compounds and ammonia emissions indicative of fugitive emissions.

This study follows a qualitative approach to classify satellite-derived emission sources into four broad categories: industry/power, transport, agriculture, and nature. The emission source categorization based on bottom-up emission inventories serves as the basis that provides a first estimation on the distribution and types of the emission sources in China. In “Emission inventories and land cover data,” we briefly describe the satellite and emission inventory data used in this study as well as the land-use dataset, followed by the development of a scheme to identify the dominant emission sources for East China (“Methodology”). With this methodology, we provide a simple satellite- and ground-based emission source categorization for the year 2010 which we intercompare and evaluate with a land cover dataset that provides independent information on the dominant land-use type responsible for emissions (“Results and discussion”). We follow the same procedure for the year 2016 to evaluate the impact of the implementation of environmental policies on the emission source classification. We put our emission source classification to the test by identifying case studies that reflect the reported reduction in the NO_x_ and SO_2_ emissions. “Conclusions” includes conclusions about our findings.

## Emission inventories and land cover data

### *Satellite-derived NO*_*x*_* and SO*_*2*_* emissions*

We use monthly top-down NO_x_ emission estimates inferred by inversion of satellite observations via the DECSO v5 algorithm (Ding et al., 2017) for the years 2010 and 2016 over East China (18–50°N, 102–132°E) as our study domain at a 0.25° × 0.25° grid resolution (www.globemission.eu). OMI provides a long-term NO_2_ tropospheric column record, and via inverse modelling, these data are used to provide top-down NO_x_ emissions, which are independent of bottom-up inventories. As input, we use OMI tropospheric NO_2_ columns from the QA4ECV (Quality Assurance for Essential Climate Variables; http://www.qa4ecv.eu/) improved NO_2_ product (Boersma et al., 2018; Zara et al., 2018). We then apply the DECSO inverse modelling system to the OMI NO_2_ data. DECSO (Mijling and van der A, 2012) is based on an extended Kalman filter with the regional chemical transport model (CTM) CHIMERE v2013 (Menut et al., 2013). This algorithm accounts for the transport of NO_x_ from its source through an isobaric trajectory analysis and takes into account the sensitivity of NO_2_ columns to local and regional NO_x_ emissions. This version 5 of the algorithm is an improvement on earlier versions for cases with weaker emission sources such as shipping emissions or small isolated sources (Ding et al., 2018). The background noise (relatively high emissions observed in remote ocean areas or low-emission areas) level above which NO_x_ emissions can be estimated with DECSO v5 applied to OMI data is 0.017 Gg N/year for a 0.25° × 0.25° grid cell (Ding et al., 2017) rendering the ΟΜΙ-derived NO_x_ emissions capable to discriminate reasonably modest signal differences.

Enhanced SO_2_ emissions allow the discrimination of highly polluting industrial areas from other emission sources such as traffic and agriculture, which usually have low SO_2_ emissions. SO_2_ emissions for China have been derived within the FP7 MarcoPolo project (http://www.marcopolo-panda.eu/). OMI SO_2_ trends have been determined on a monthly basis for each Chinese province for the period 2005–2018 (Theys et al., 2015). These trends are used to scale SO_2_ emissions from the MIX emission inventory (Li et al., 2017) for the reference year 2010 at a resolution of 0.25° × 0.25° and provide OMI-consistent SO_2_ emission estimates for our study period 2010–2016.

Apart from anthropogenic and natural NO_x_ emission sources (discussed in “Bottom-up NO_x_ and SO_2_ emissions from sectors”), crop residue fires cause air pollution and harmful health effects on an episodic basis. Crop residue burning has distinct seasonal and spatial variability throughout East China with the most intense burning occurring in the North China Plain (32–40°N, 112.5–120°E) in the month of June (Stavrakou et al., 2016). These fires emit significant amounts of NO_x_, which potentially hampers the classification of the emission sources during that month. Fire-induced enhanced CO emissions help to identify and then to remove from the annual estimate the NO_x_ signal originating from June crop fires (for more details see Table S1; Fig. S3). Here, we use CO emission estimates inferred from OMI QA4ECV formaldehyde (HCHO) columns and a flux inversion scheme based on the discrete adjoint model of the IMAGESv2 global CTM (Stavrakou et al., 2016). As reported in that study, the top-down crop burning fluxes of VOCs in June (735 Gg VOC) exceed by almost a factor of 2 the combined emissions from other anthropogenic activities in the North China Plain in that month underlining their substantial contribution to the poor summertime air quality in this region. This post-harvest burning releases 25 Gg NO_x_ (up to 50 Gg NO_x_, expressed as N, considering up-to-date emission factors) comprising the 12% (up to 22%) of the total anthropogenic (crop residue fires and anthropogenic emissions from other sources) load in North China Plain in June.

### *Bottom-up NO*_*x*_* and SO*_*2*_* emissions*

Bottom-up SO_2_ and NO_x_ emissions in East China are taken from the MIX inventory (Li et al., 2017; http://www.meicmodel.org/dataset-mix). This inventory was developed for the years 2008 and 2010 (with monthly resolution) in support of the Model Inter-Comparison Study for Asia (MICS-Asia) and the Task Force on Hemispheric Transport of Air Pollution (TF HTAP) from a mosaic of up-to-date regional emission inventories. Emissions are estimated for all major anthropogenic sources in 29 countries and regions in Asia at a 0.25° × 0.25° grid resolution by incorporating the best available emission inventories for each region into the inventory at a uniform spatial and temporal resolution. Emissions are aggregated from four anthropogenic sectors: industry, power generation, transportation, and residence.

Additionally, we use the Regional Emission inventory in ASia (REAS) v2.1 (Kurokawa et al., 2013; http://www.nies.go.jp/REAS/) for agricultural NO_x_ emission estimates (not included in MIX) in 2008. Agricultural emissions remain relatively constant throughout the years (e.g., Weng et al, 2020; Zheng et al., 2018), and therefore, we assume that the 2008-REAS agricultural emission estimates stand for 2010 as well. REAS v2.1 is an improvement over REAS v1.1 via improved basic activity data, parameters, and methodologies.

### *Land use data*

We evaluate our emission categorization scheme with independent land-use data from the European Space Agency (ESA) GlobCover Portal (http://due.esrin.esa.int/page_globcover.php). The GlobCover project developed land cover maps using 300 m MERIS data as input observations. We regridded the 2009 GlobCover data (0.0083° resolution) to 0.25° × 0.25° to match the satellite- and inventory-based emission classification for a straightforward comparison. GlobCover has been extensively validated and compared to other available land cover products (e.g., Hua et al., 2018; Pérez-Hoyos et al., 2012; Verhegghen & Defourny, 2010). It was found that land cover classes such as evergreen and semi-deciduous forest, irrigated croplands, bare areas, water bodies, and snow-covered areas were accurately mapped in GlobCover. Other classes such as urban areas, sparse vegetation, and herbaceous vegetation are more affected by errors (Hua et al., 2018; Verhegghen & Defourny, 2010), but we deem the quality of the land use data sufficient to serve as an independent means of comparison to our dominant-emission-source classification.

## Methodology

### *Bottom-up NO*_*x*_* and SO*_*2*_* emissions from sectors*

**Fig. 1:**
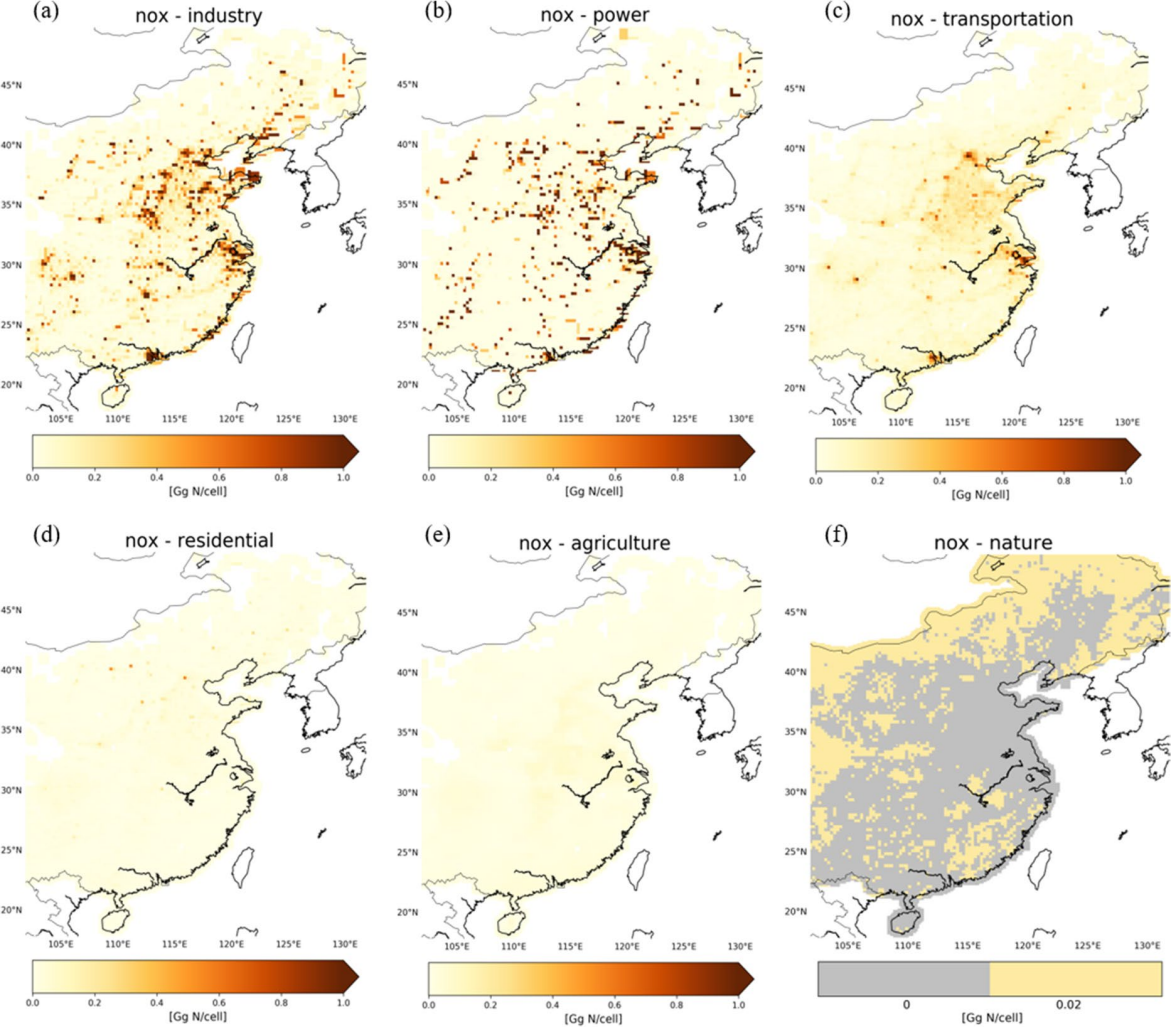
**a**–**e** Bottom-up NO_x_ emissions from the SMIX inventory (industry, power, transportation, residential) and the REAS v2.1 inventory (agriculture: livestock and fertilizer application) for East China in 2010 on a 0.125° × 0.125° grid. **f** Spatial distribution of natural soil NO_x_ emissions of 0.02 Gg N/cell/year

**Fig. 2:**
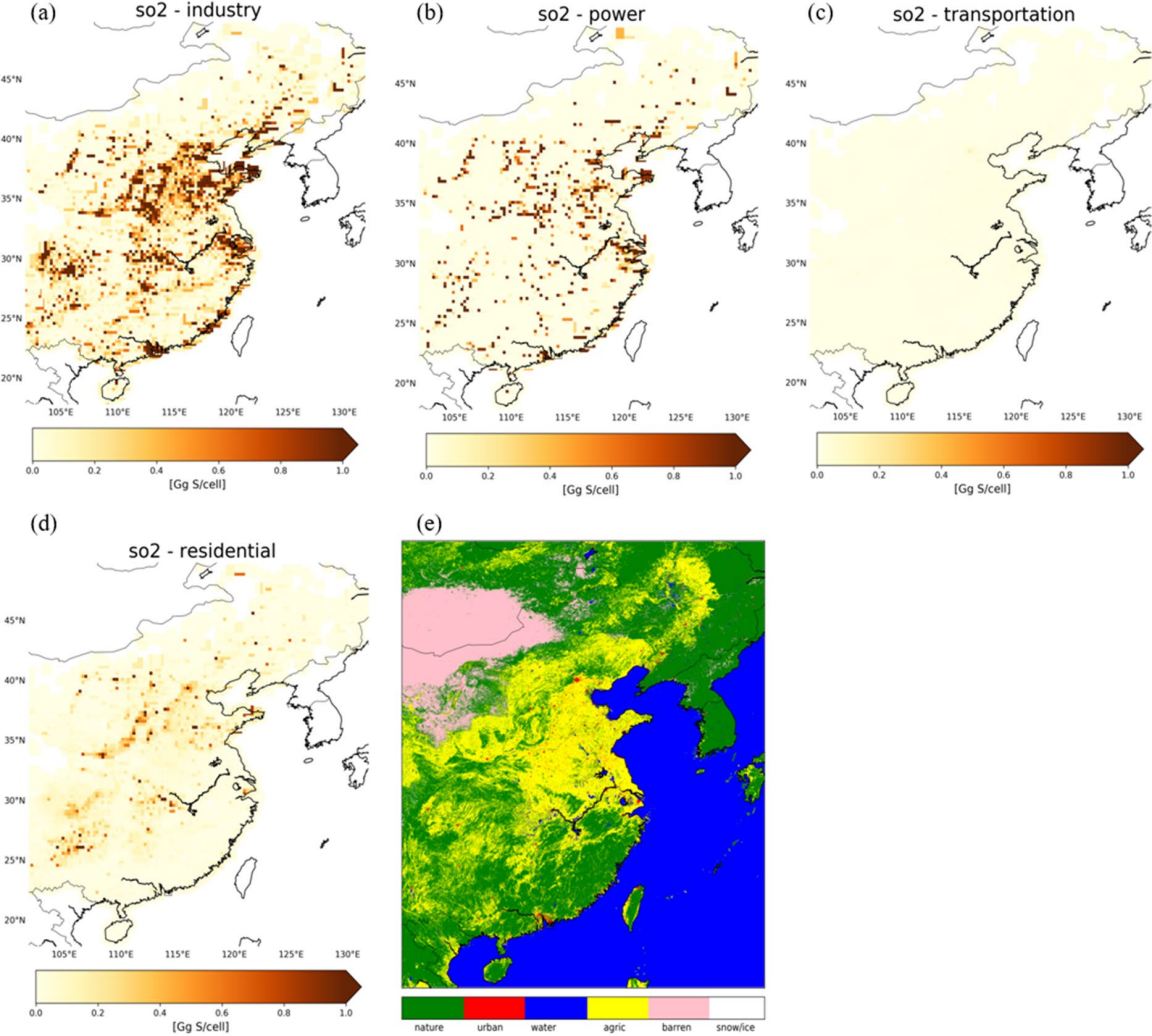
**a**–**d** Bottom-up SO_2_ emissions from the SMIX inventory (industry, power, transportation, residential) for East China in 2010 on a 0.125° × 0.125° grid. **e** Land-use data based on GlobCover for the year of 2009 on a ~ 0.00833° × 0.00833° grid. Six land-use categories are displayed: nature, urban, water, agriculture, barren, and snow/ice as determined in “Land use data” and “Comparison with land cover data.”

Our analysis focuses on 2010, and the heavily polluted eastern part of China. Figures 1 and 2 show NO_x_ and SO_2_ emissions from the residential, industrial, power, and transportation categories in the SMIX^1^ inventory (i.e., the MIX inventory supplemented with the natural and agricultural soil NO_x_ contributions; see below). The strongest NO_x_ emissions are from combustion processes by industry (~ 2.9 Tg N/year), power generation (~ 2.4 Tg N/year), and transportation (~ 1.7 Tg N/year) (Fig. 1a–c). Residential NO_x_ emissions are relatively weak (~ 0.3 Tg N/year), and their patterns generally coincide with NO_x_ emissions from transportation (Fig. 1d). For SO_2_, the strongest emissions originate from industry (~ 7.4 Tg S/year) and power generation (~ 3.6 Tg S/year), followed by residential emissions (~ 1.5 Tg S/year) (Fig. 2a–b, d), and transportation (~ 0.1 Tg S/year) (Fig. 2c).

The MIX inventory provides no information about soil NO_x_ emissions from agricultural activities and natural microbial processes (SO_2_ emissions from such sources are negligible; EEA 2020-LRTAP Convention). We therefore include agricultural soil NO_x_ emissions (livestock and fertilizer application) of 0.5 Tg N/year for East China from the REASv2.1 inventory for 2010 (Fig. 1e). These emissions are lower than industrial and transportation sources, but still relevant, and mostly occur in the growing season.

Previous studies (Wang et al, 2004, 2007; Jaeglé et al., 2005; Lin et al., 2012) estimated soil NO_x_ emissions to be in the range of 0.4 to 1.1 Tg N/year for East China. Here, we take 0.85 Tg N/year as representative estimate for natural and agricultural NO_x_ sources combined (Wang et al., 2007) for East China. Such emissions remain relatively constant from year-to-year (e.g., Weng et al, 2020; Zheng et al., 2018). From this estimate, we subtract the REAS agricultural soil NO_x_ emissions (0.5 Tg N/year) to arrive at a natural soil NO_x_ emission estimate of ~ 0.35 Tg N/year. Land-cover data provides an independent insight into the distribution of barren areas, low-vegetation and grasslands, forests, water bodies, and agricultural and urban regions across China (Fig. 2e). According to the land-use data, approximately half of the land of East China is considered to be nature (~ 45%) or barren (~ 7%) to which we attribute ~ 0.35 Tg N/year of natural soil NO_x_ emissions. This translates into ~ 0.02 Gg N/year per grid cell of natural (forests, grasslands, shrublands, deserts) NO_x_ soil emissions for East China in 2010 (Fig. 1f). Another natural source of NO_x_ to the atmosphere in summertime is NO_x_ production from lightning. The strength of lightning NO_x_ production is estimated to be ~ 0.2 Tg N over East China (Lin, 2012). These emissions contribute a substantial amount of NO_x_ to the upper troposphere (e.g., Boersma et al., 2005; Zhang et al., 2020) and will be omitted from this analysis because they mostly affect tropospheric background NO_2_ levels and the focus here is on emissions at ground level.

### *Identification of the dominant emission categories*

This study follows a qualitative approach to classify satellite-derived emission sources in East China into four broad categories: industry/power, transport, agriculture, and nature. We use the emission source categorization based on bottom-up emission inventories as a first estimation on the distribution and types of the emission sources in this region. To identify the bottom-up dominant emission sources, we explore the SMIX inventory in three main steps:

- Step 1: Identification of the dominant emission category in each SMIX grid cell,
- Step 2: Determination of quantitative separation points (hereafter “limits”) that distinguish emission categories via a cross-evaluation of the SMIX NO_x_ emissions and corresponding SO_2_:NO_x_ ratio,
- Step 3: Application of the limits to discriminate between emission categories in the OMI NO_x_ emissions and their SO_2_:NO_x_ emission ratio.

Below we describe each step in detail.

Each SMIX grid cell is characterized by contributions to the overall NO_x_ and SO_2_ emissions from the categories industry, power generation, transportation, residence, agriculture, and natural soils. Our scheme uses the absolute and relative strength of the SMIX NO_x_ and SO_2_ emissions, which differ substantially between the various source categories. For example, industry and power generation are generally characterized by high NO_x_ and SO_2_ emissions. Transportation and the residential sector are known for their high NO_x_ emissions and relatively low SO_2_ emissions, while agricultural activities release modest amounts of NO_x_ emissions that peak in the growing season and almost no SO_2_ emissions.

**Fig. 3:**
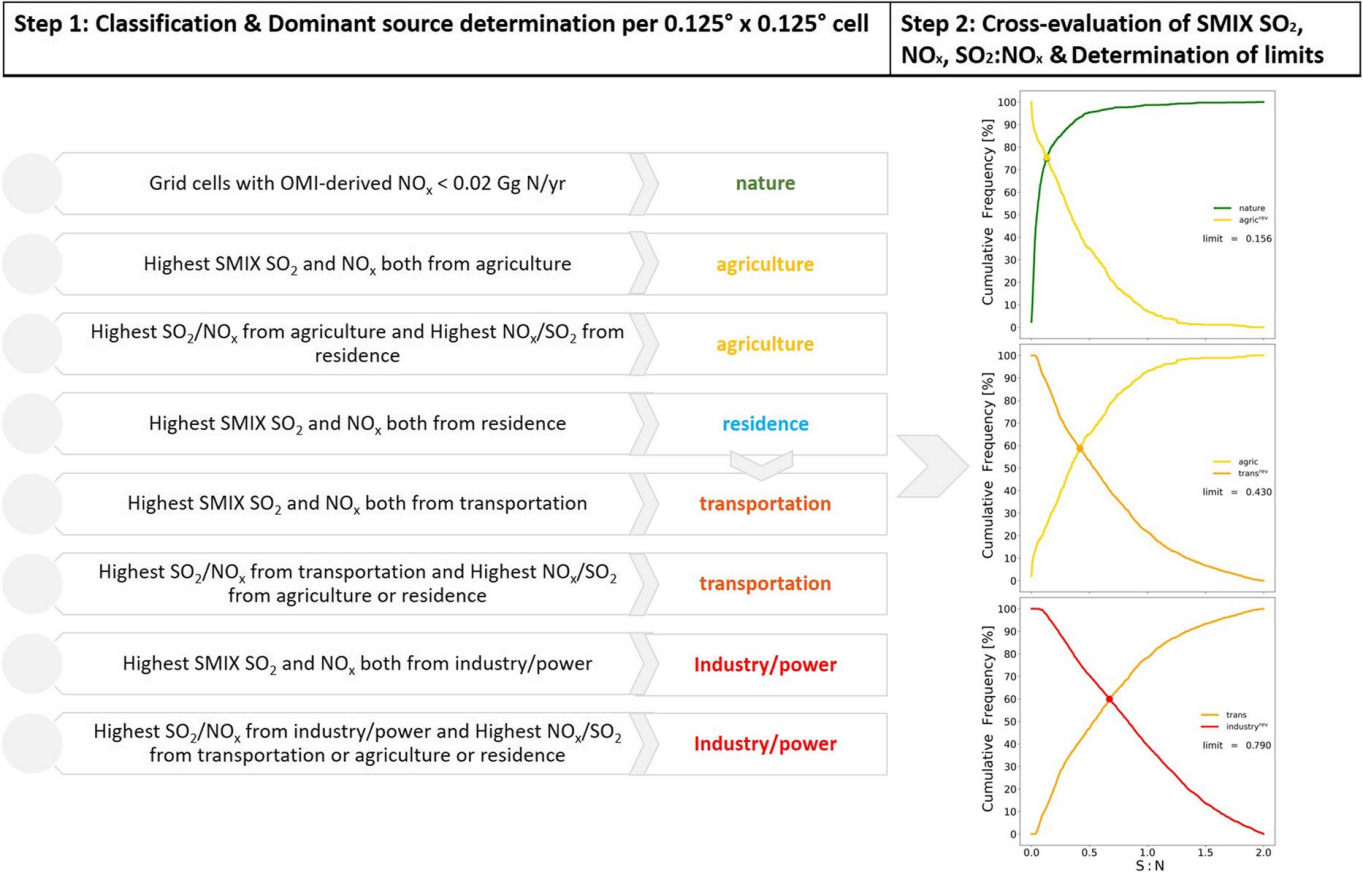
Flowchart of the steps taken after the pre-classification to detect the transitioning points between emission categories based on SMIX SO_2_ (as S) and NO_x_ (as N) emissions and their SO_2_:NO_x_ (as S:N) ratio for East China (2010). The meeting point of the SO_2_:NO_x_ cumulative frequencies of two emission categories denotes their transition point (“limit”). The cumulative frequency of the category with the higher emission strength is shown reversed (i.e., from 100 to 0%). The number of occurrences where the highest SO_2_ and NO_x_ emissions are both originated from residential sources is negligible (0.01%); therefore, the residence category is merged with transportation. These limits are later applied to the OMI-derived SO_2_ and NO_x_ emissions to determine the satellite-based emission categories. See text for more context

Figure 3 shows the categorization scheme applied to each grid cell. Step 1 begins with grid cells with OMI NO_x_ emissions < 0.02 Gg N/cell/year to be identified as “nature” as inferred in “Bottom-up NO_x_ and SO_2_ emissions from sectors.” The location of these cells is carried onto the SMIX inventory and defines the SMIX “nature” emission category. The remaining grid cells then undergo a check regarding their SMIX NO_x_ and SO_2_ emission strength. We merge the “industry” and “power” categories into one, as they both have high NO_x_ and SO_2_ emissions originating from fossil fuel combustion, especially coal burning. For each grid cell, we retain the category that reports the highest NO_x_ emissions and the category that reports the highest SO_2_ emissions (these are not necessarily the same). If the categories for the highest NO_x_ and highest SO_2_ emissions are identical, the single (dominant) emission source category for that grid cell is established. If this is not the case, the category that is most important for air pollution in China is prioritized and chosen as the dominant emission source. The categories are prioritized in the following order: industry/power > transportation > agriculture > residence. This is in line with Li et al. (2017) who report anthropogenic emission estimates by sectors compared to the total anthropogenic emissions in China: industry/power (NO_x_: 68%; SO_2_: 84%), transportation (NO_x_: 26%; SO_2_: 0.8%), and residence (NO_x_: 6%; SO_2_: 15%). We find that the number of occurrences (i.e., grid cells) where the highest NO_x_ and SO_2_ emissions are both originating from residential sources is extremely low (0.01%), and therefore, we merge the residence category with the transportation category since transportation is strongly associated with populated areas.

Our scheme indicates that much of East China’s pollution was characterized by industrial and power sector emissions in 2010 (Fig. S1). Step 2 analyses the distribution of SMIX NO_x_ and SO_2_ emission strengths for each category (Figs. 3 and S2). We cross-evaluate emission categories based on their ascending emission strength; we compare nature with agriculture to find the most representative emission strength below which emissions can be considered to originate from natural sources. We then compare agriculture with transportation to establish the value below which emissions are still typical from agriculture, and so on. Table 1 shows the meeting point of the SO_2_:NO_x_ cumulative frequencies of the two emission categories that establish the transition point or “limit” between these categories.

**Table 1:** Transitioning points (‘limits’) between neighbouring emission categories as determined in the scheme displayed in Figs. 3 and S2

*Cross-checked emission categories*	SMIX NO_x_ limits [as Gg N/cell/yr]	SMIX SO_2_:NO_x_ limits [as S:N per cell/yr]
Nature	< 0.030	< 0.156
Agriculture	0.030–0.045	0.156–0.430
Transportation	0.045–0.083	0.430–0.790
Industry	> 0.083	> 0.790

Finally, in step 3 we apply these limits to OMI NO_x_ and SO_2_ emissions to classify the satellite-derived emissions of NO_x_, SO_2_, and their SO_2_:NO_x_ into one dominant category. To avoid interference from NO_x_ emitted from crop fires that dominate North China Plain in June we remove this month from the annual NO_x_ emission estimate for grid cells with CO emissions higher than 0.5 Gg CO/cell/year for June. The choice of the threshold value is discussed further in the Supplementary Material (Table S1; Fig. S3).

## Results and discussion

### *Comparison of OMI and SMIX dominant emission sources*

**Fig. 4:**
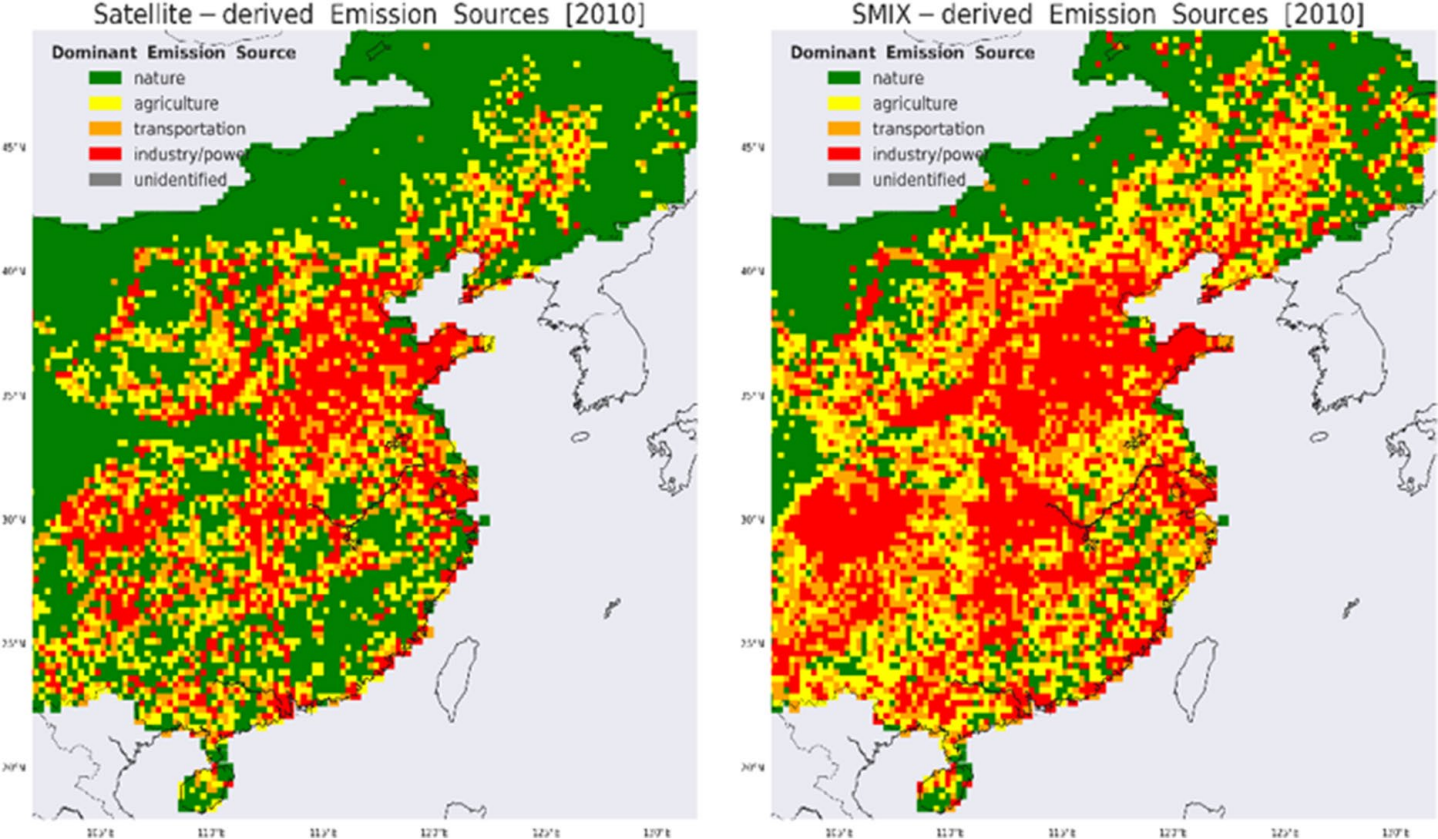
(left) Satellite-derived (dominant) emission source categorization based on OMI NO_x_ and SO_2_ emissions for East China in 2010 using the limits determined in Table 1; (right) same as left but for the SMIX NO_x_ and SO_2_ emissions. The spatial resolution is 0.25° × 0.25°

We apply the limits that distinguish the emission categories (Table 1) to the OMI-based and SMIX NO_x_ and SO_2_ emissions. Figure 4 displays the dominant emission source classification based on satellite-derived NO_x_ emissions and SO_2_:NO_x_ ratio in 2010 and after the application of the same limits to the bottom-up SMIX NO_x_ and SO_2_ emissions, and their ratio, for comparison. Some of the largest agglomerations of industry/transportation clusters are located in the eastern part of East China in the provinces of Jiangsu, Shanghai, Zhejiang, Fujian, and Guangdong, areas where the GDP per capita is generally higher than the national average (Wang & Mei, 2009), also well represented by both OMI and SMIX. Following the “Go West” stream that aims at favouring the development of the central provinces of China, more clusters are developed in those areas: Shanxi, Shaanxi, Hubei, Henan in the west with Hebei, and Shandong in the north.

While the spatial distribution of the heavy industrialized areas, large cities, agricultural lands, and the natural (or barren) landscape shows good similarity between SMIX and OMI, the OMI-based classification shows generally fewer cells dominated by industry/power or by agriculture, and more cells dominated by nature. The satellite-based emission categorization is less dominated by the industrial footprint (18% of the cells are classified as industry/power) compared to SMIX (31%). For the sectors of transportation (OMI: 12%; SMIX: 18%) and agriculture (OMI: 16%; SMIX: 21%), the shares are comparable. The fraction of natural scenes is distinctly higher in the satellite classification (OMI: 55%; SMIX: 30%). Roughly half of the cells classified by OMI as nature are categorized by SMIX as cells dominated by agriculture (25%), transportation (15%), or industrial (11%) emissions. In addition, 13%, 17%, and 19% of the SMIX industrialized areas are classified as agricultural, transportation, and natural emission sources by OMI.

Compared to the bottom-up emission categorization, the satellite perspective suggests an emission landscape less dominated by industrial areas, a somewhat larger role for transportation/urban areas and agricultural activities in East China, and more natural areas in the southeastern part of China. The main reason is that the satellite-based NO_x_ load is ~ 33% lower than SMIX NO_x_ over East China in 2010 (~ 5.7 Tg N vs ~ 8.5 Tg N; SO_2_ emissions are comparable with ~ 13.2 Tg S; see “Satellite-derived NO_x_ and SO_2_ emissions”). Other plausible sources of the discrepancies between the satellite and emission inventory records could be (1) the large footprint of the OMI pixel compared to the finer resolution of bottom-up inventories and (2) the larger contribution of background NO_2_ in the NO_2_ columns observed by OMI in rural areas which are used in the inverse modeling technique to infer satellite-based emissions and would draw a “greener” picture from OMI (e.g., Zara et al., 2021), (3) the threshold of ~ 600 MW of the power plants emission strength detected by OMI (Yan & Xu, 2021) that would indicate a landscape less dominated by industry.

### *Comparison with land cover data*

**Fig. 5:**
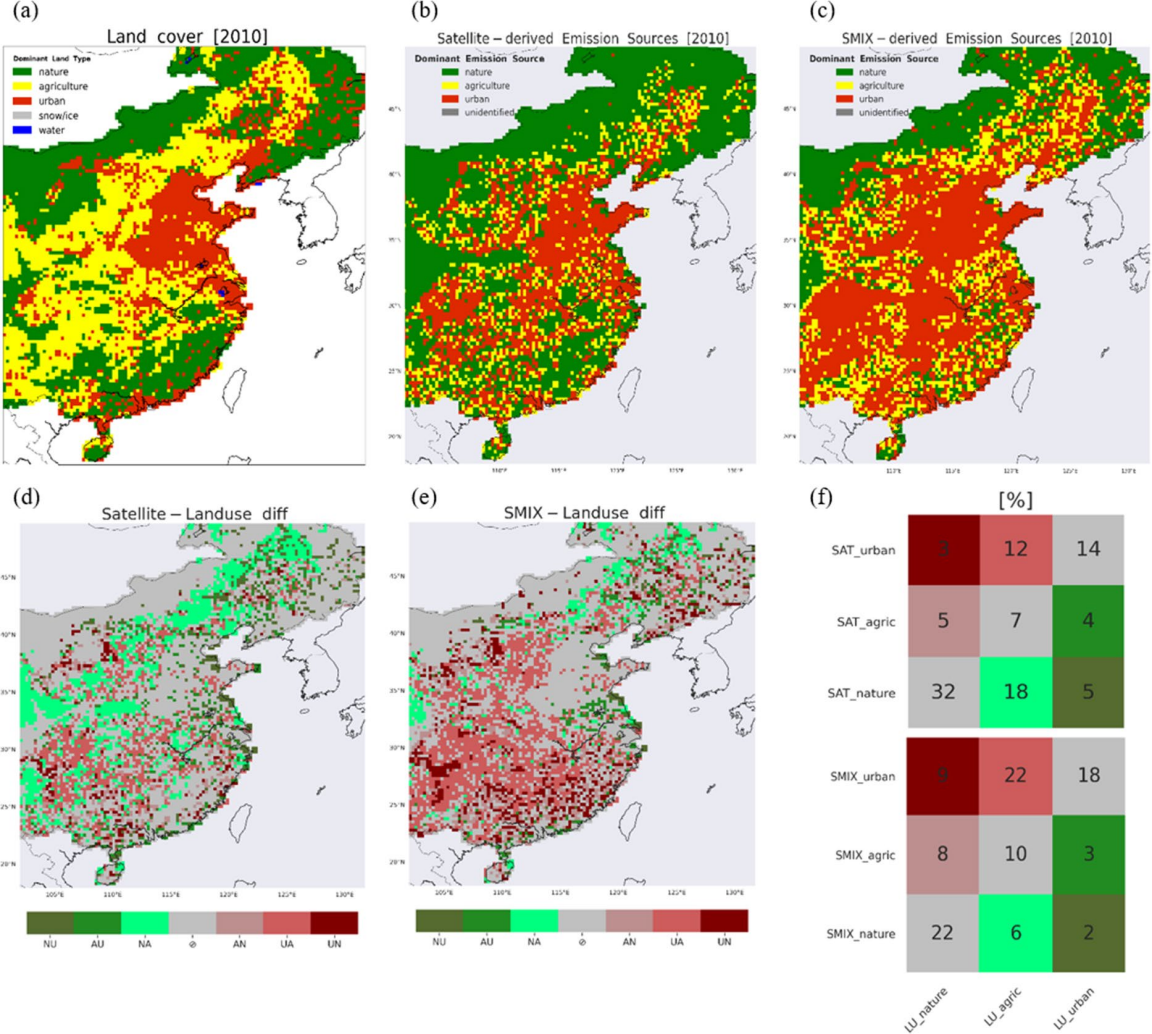
**a** GlobCover (dominant) land type. The category “bare areas” has been integrated into the nature category. The spatial resolution is 0.25° × 0.25°. **b**–**c** Satellite- and SMIX-derived (dominant) emission source categorization (same as Fig. 4) with the transportation and industry categories merged into the broad urban category. **d**–**e** Difference between the OMI/SMIX-derived and GlobCover-derived dominant emission source classification. Each colour corresponds to each case of emission classification disagreement between the datasets; e.g. ‘NU’ (olive green) denotes the case where OMI/SMIX characterizes the cell as nature dominated and land use sees it as urban dominated. Similarly, ‘NA’: nature to agriculture, ‘AU’: agriculture to urban, ‘AN’: agriculture to nature, ‘UA’: urban to agriculture, ‘UN’: urban to nature. The grey colour refers to agreement between OMI/SMIX and GlobCover emission classification. **f** Occurrences (shown as percentages) of all cases of agreement and disagreement between OMI/SMIX and GlobCover dominant emission categorization for the three types of emission categories: nature, agriculture, urban. See text for more context

We compare our dominant emission source categories from OMI and SMIX with the independent land-cover information from the GlobCover dataset. The GlobCover provides gridded information on the dominant type of land use at a resolution of ~ 0.00833° (Fig. 2e). This is much finer than our satellite- and inventory-based emission categorization with a resolution of 0.25°, so we first perform a generic reclassification and a series of checks to establish the dominant emission source in the GlobCover database on the coarser grid for a 1:1 comparison against OMI and SMIX. The same principle applies to the 23 land-use categories that comprise the GlobCover dataset (Table S2). To compare against OMI and SMIX, we merge land-use categories of similar type into the same broad categories seen in OMI and SMIX: GlobCover categories 1–3 are of agriculture-type, comparable to the agriculture category from OMI and SMIX; categories 4–18 are of nature-type, comparable to the nature category from OMI and SMIX; category 19 is classified as urban, comparable to the industry and transportation categories from OMI and SMIX; and category 20 is of barren-type, categories 21 and 23 are of water-type, and category 22 is of permanent snow/ice-type, comparable to the nature category in OMI and SMIX. The result of this reclassification is shown in Fig. 2e, but still at the fine resolution of ~ 0.00833°. To arrive at a dominant GlobCover emission category over China at a 0.25° × 0.25° spatial resolution, we need to establish the dominant emission land type within each 0.25° grid cell. If at least 1% of the area of a 0.25° cell is classified as “urban” in GlobCover, we classify the entire cell as “urban,” because urban NO_x_ and SO_2_ emissions are generally much (at least a factor of 10) (e.g., Wang et al., 2004; Jaeglé et al., 2005; Martin et al., 2006; Wang et al., 2007; Jyethi, 2016; Kang et al., 2019) stronger than emissions from agriculture or nature. This serves as a top priority check. We classify a cell as “agriculture,” if 25% of the cell is classified in GlobCover as agriculture (and the remainder as nature). The 1% and 25% coverage thresholds are motivated by intense anthropogenic activities such as industry and transportation having much larger emissions than other sources. A small industrial footprint outweighs spatially more spread-out but weaker agricultural or natural emissions. The same reasoning holds for agricultural land surrounded by natural areas. Figure 5 a shows the result of our reclassification of land-use types into 0.25° emission categories for East China in 2010.

The GlobCover data do not distinguish between industry/power and transportation as dominant categories, but only provide the urban category to represent the strong anthropogenic stigma in air pollution. To be able to compare to the GlobCover “urban” category, we merge our satellite/SMIX “transportation” and “industry” categories into one single category named “urban.” This now allows us to compare the three different categories “urban,” “agriculture,” and “nature” between the GlobCover, OMI-based, and SMIX-based classifications. Figure 5 compares the spatial distribution of the GlobCover, OMI-based, and SMIX-based emission classifications over East China. We find that OMI and SMIX classifications agree on the dominant emission source with GlobCover for half of our domain (53% and 50%), mostly in areas with a strong presence of industry (North China Plain and its surroundings) or nature (far from main cities and agricultural activities), as indicated by the grey cells in Fig. 5d–e. The most prominent differences between the OMI and GlobCover classifications occur over the northern and western parts of the domain classified as nature by OMI but as agriculture by GlobCover (light green cells in Fig. 5d), and in the southwestern part of the domain where OMI reports urban rather than agriculture as the dominant source (red cells in Fig. 5d). Compared to GlobCover, SMIX mainly classifies cells throughout the southwesterly half of the domain as dominated by urban rather than agriculture (red in Fig. 5e) and rather than nature (dark red in Fig. 5e) sources. Vast agricultural lands mixed with patches of nature and small industrial sites are more distinctly picked-up by the satellite and inventory rather than the GlobCover database.

### *Impact of emission reduction measures on the emission source type*

**Fig. 6:**
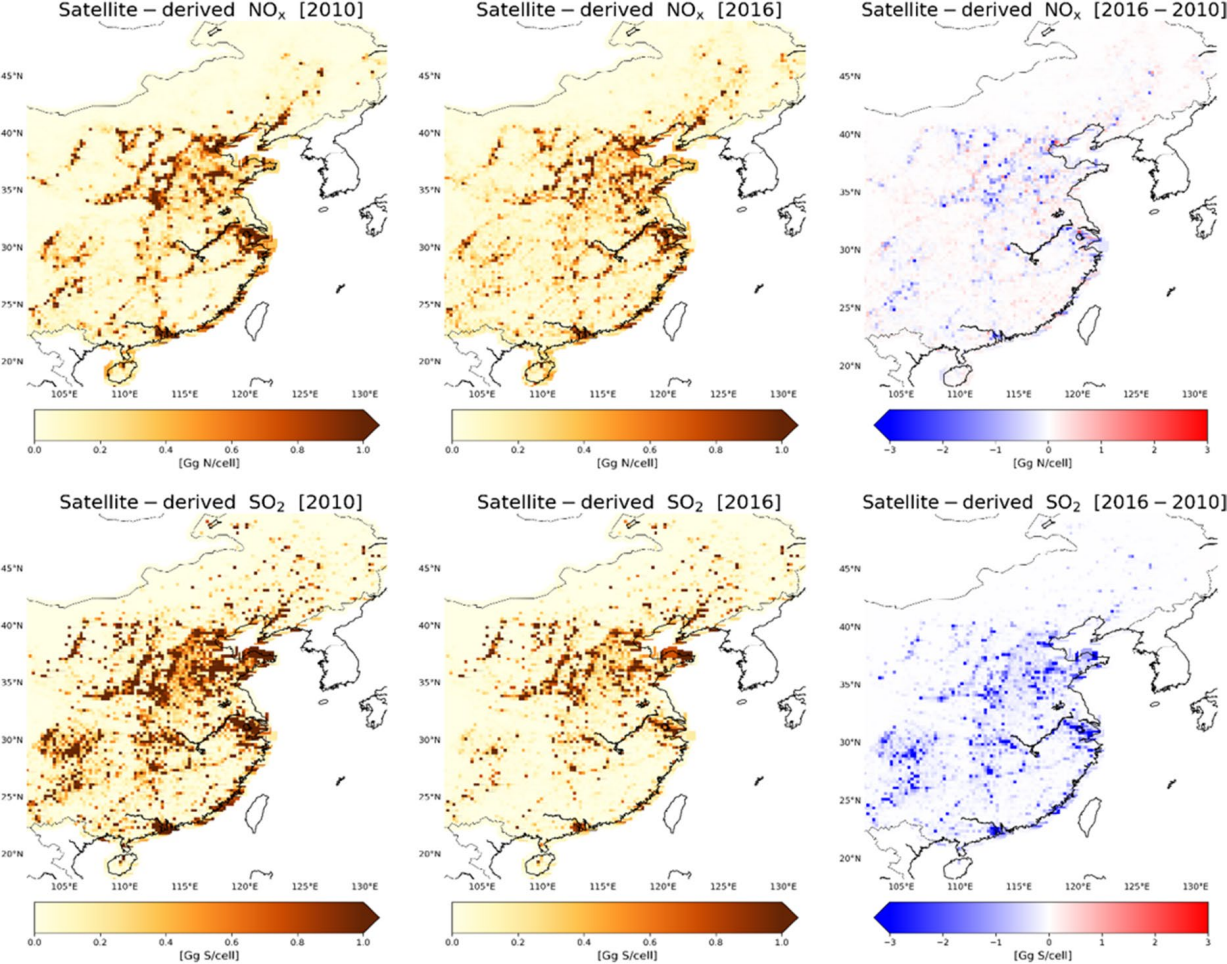
Satellite-derived NO_x_ and SO_2_ emissions in 2010 (left) and 2016 (middle), and their absolute differences (right) in East China. The spatial resolution is 0.125° × 0.125°

The satellite-derived emissions in East China in 2010 are estimated at ~ 5.7 Tg N and ~ 13.2 Tg S (Fig. 6). NO_x_ emissions peak in 2012 with an increase of 14% compared to 2010 (Fig. S4) and start to decrease in 2013, when China’s Clean Air Action was enacted and implemented. SO_2_ emissions decrease from 2011 onwards. In 2016, satellite-derived NO_x_ and SO_2_ emissions in East China dropped by ~ 8% and ~ 58% compared to 2010, respectively, indicating the effectiveness of the governmental measures to reduce air pollution.

**Fig. 7:**
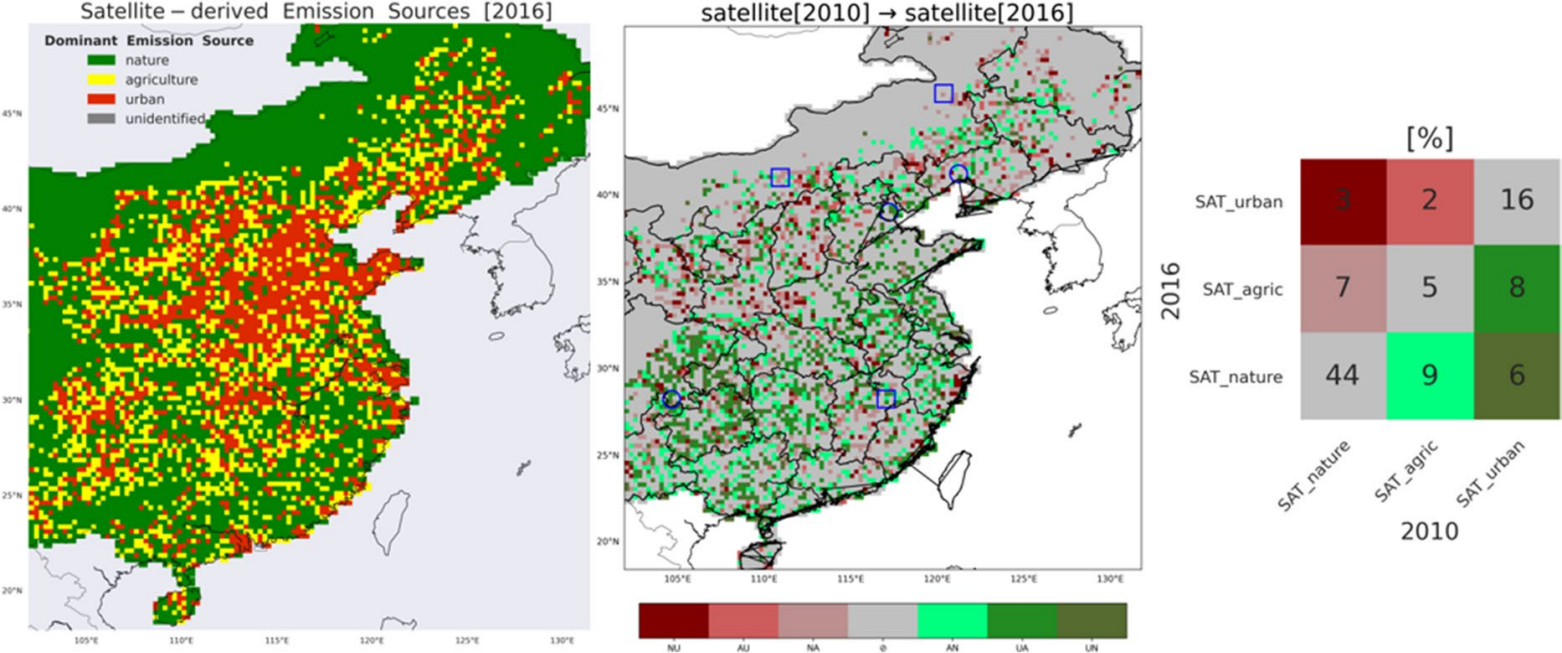
(left) Satellite-derived (dominant) emission source categorization (same as Fig. 5b) but for 2016. (middle) Transition of the OMI-derived dominant emission source classification from 2010 to 2016. Each colour corresponds to a case of emission classification change between the 2 years; e.g. ‘NU’ (maroon) denotes the case where 2010 OMI characterizes the cell as nature-dominated and 2016 OMI sees it as urban-dominated. Similarly, ‘NA’: nature to agriculture, ‘AU’: agriculture to urban, ‘AN’: agriculture to nature, ‘UA’: urban to agriculture, ‘UN’: urban to nature. The grey colour applies to an agreement between 2010 and 2016 OMI emission source classification. New and closed coal-fired power plants are denoted with blue squares and circles, respectively. See text for more context. (right) Occurrences (shown as percentages) of all cases of transition between 2010 and 2016 OMI-derived dominant emission categorization for the three types of emission categories: nature, agriculture, and urban. See text for more context. The spatial resolution is 0.25° × 0.25°

We now evaluate whether the reduction in the NO_x_ and SO_2_ emissions is reflected by changes in our emission source classification. Applying the limits shown in Table 1 to the satellite-derived NO_x_ and SO_2_ emissions in 2016 (Fig. 7), we find an overall decrease in heavy air pollution in East China in the span of seven years (Fig. 7, middle). Our scheme classifies 59% of East China as “nature,” a net increase of 5% relative to 2010. At the same time, there is a 4% increase of agricultural lands and a 9% decrease of urban (industry- and transport-dominated) cells in 2016 relative to 2010. These changes also appear as a general movement of industry and transportation from East to North and North-West, also supported by the “Go-West” stream. Approximately 65% of the emissions classified over East China have remained the same between 2010 and 2016 (grey areas). Overall, the share of urban areas has decreased from 30 to 21%, and has mostly shifted to the less polluting category of “agriculture” (8%). The most notable reduction of air pollution has taken place in regions in the south (including the Pearl River Delta and the Yangtze River Delta), adjacent to areas that were already classified as nature in 2010. Over the North China plain, several areas have successfully scaled down their heavy emissions, especially in the densely populated eastern part (green). The provinces of Jilin and Heilongjiang (northeast) and the provinces of Ningxia and Shaanxi (northwest) reveal a strong transition to more intense anthropogenic activities (red). The Shanghai province shows a 1–2% decrease in urbanization in 2016 relative to 2010 (China Statistical Yearbook [2017]), also depicted in Fig. 7.

As expected, the noticeable rural–urban migration coupled with the accelerated population growth is associated with the environmental degradation observed in the urban areas (urban expansion), but more strongly with NO_2_ and PM_10_ pollution and has a weaker relationship with SO_2_ (Qin & Liao, 2016). The urban population growth in China resulted to stronger emissions from sources representing consumption and transportation domains (e.g. NO_x_) which are directly influenced by the population size. The same study suggests that the rural–urban migration is more influential than nominal urban increase for the environmental impacts of population dynamics in China and possibly other rapidly urbanizing developing countries, such as China. The rural–urban migration contributes to ecosystem recovery and conservation in origin areas but has severe effects on environmental conditions in the migration destinations. For instance, according to the China Statistical Yearbook [2017], the fraction of the total population living in cities in the Jilin and Heilongjiang provinces has increased by 2.6% and 3.5%, respectively, between 2010 and 2016. The urbanization is stronger in the provinces of Ningxia and Shaanxi (8.4% and 9.6%, respectively). These statistics are also evident in Fig. 7 (middle). Overall, 12% of previously flagged as areas with relatively low emissions have transitioned into areas with heavier pollution (mostly in the North and Northwest), while 23% of previously flagged as areas with relatively high emissions have shifted into cleaner regions (mostly in the East).

At the same time, air pollution has driven population migration to areas with cleaner air. Zhao et al. (2021) report that factors such as gender, age, education, and financial status can stimulate population movement. Male citizens are more sensitive to air pollution due to their higher expose to industrial equipment and automobile exhaust, and less keen to settle in polluted areas. Age is an essential demographic feature, intertwined with the educational background and financial status thereof: young population, especially with high degrees of education, tends to pay more attention to employment opportunities and is likely to reside in big cities to pursue a career and reap certain economic benefits at the cost of poorer air quality. As this floating population enters the middle-aged and elderly phases of life, these individuals are more likely to seek a better quality of life and physical health, and now being able to afford the costs of migrating, they are motivated to relocate in cleaner regions.

Our results suggest that the strong reduction in SO_2_ emissions (~ 58%) and smaller but still substantial reduction in NO_x_ emissions (~ 8%), primarily because of the applied environmental policies, have shifted urban areas into less polluted areas (e.g., agriculture and nature) between 2010 and 2016. This could point at industrial sites having been shut down or relocated classifying them in the agricultural or natural categories in 2016. Indeed, we find that the locations of three major coal-fired power plants that closed down between 2010 and 2016 coincide with a transition from urban to cleaner categories (UA: urban to agriculture, UN: urban to nature) in the OMI-based categorization (Fig. 7, middle): (1) the Jinzhou CR Power Station (41.27°N, 121.25°E) in Liaoning Province with a capacity of 1320 MW retired in 2014 (NE blue circle); (2) the Chentangzhuang Power Station (39.05°N, 117.24°E) in Hexi, Tianjin Municipality, with a capacity of 920 MW closed by 2015 (replaced by a 1.800-MW gas-fired power plant) (Center-North blue circle); and (3) the Gongxian Power Station (28.27°N, 104.67°E) in Sichuan Province with a capacity of 1200 MW shut down in 2014 over its high emissions (SW blue circle). The termination of these coal-fired power stations is reflected in a transition from 2010 industry/transportation dominated cells to cleaner areas in 2016 (Fig. 7).

On the other hand, cities-clusters appear to have sprawled into previously nature-dominated areas and are dominated in 2016 by urban and agricultural activities (housing, transportation, industry). This is in an agreement with Chu [2020] who reported large cities’ growth (with exception the Pearl River Delta and the Yangtze River Delta), and population movement to inland and the western part of the domain. This hints for people’s effort to abandon large and heavily polluted city centres and move to their outskirts (urban expansion), which in combination with the rural–urban migration motion, the emission budget for East China in 2016 either appears unchanged (grey) or increased (red) (Fig. 7). Provinces such as Jangxi in the south, Shanxi and Shaanxi in the centre, and Beijing, Ningxia, and Inner Mongolia in the north of East China show strong indications of urban expansion from 2010 to 2016.

Some major coal-fired plants with heavy capacity have opened between 2010 and 2016, and our categorization scheme indeed confirmed that their location corresponds with a transition to a more polluted scene (NU: nature to urban, AU: agriculture to urban) in 2016 (Fig. 7, middle): (1) the Datang Fuzhou Power Station (27.79°N, 116.56°E) in Jiangxi Province with a capacity of 2000 MW whose Unit 1 was completed in August 2015, and unit 2 in April 2016 (South blue square), (2) the Baotou Aluminum Power Station (40.56°N, 110.15°E) in Inner Mongolia Autonomous Region with a capacity of 1710 MW was put in operation in June 2014 (NW blue square), and (3) the Huolinhe Zhanute II Power Station (45.41°N, 119.59°E) in Inner Mongolia with a capacity of 1.320 MW whose units 3 and 4 were completed in winter 2015 (North blue square). Overall, the effectiveness of the applied governmental environmental policies is undeniable yet the air quality in East China remains a concern.

## Conclusions

This study generates a space-based qualitative emission source characterization for East China in the period 2010–2016. It is focused on the heavily polluted eastern part of China where environmental policies have changed over the years allowing to assess the impact of the new measures from the satellite perspective but also from bottom-up emission inventories. The source identification technique developed in this work uses the satellite NO_x_ and SO_2_ emissions, and their SO_2_:NO_x_ ratio, as an indicator to distinguish between different emission regimes.

Given the complexity of the spatial distribution and composition of the emission sources, in reality a region is a mix of different types of emission sources (a region’s emission load can be driven by e.g. both industry and transportation) for which we already accept collective emissions (e.g., NO_x_ emissions were not discriminated further). We identify the dominant emission source per cell using three to four broad categories in total: a required simplification for the direct comparison of bottom-up emission inventory-, satellite-, and land use-based emissions provided at different spatial resolutions and emission categories. These simplifications accommodate an elementary yet independent, reproducible, and qualitative satellite–based emission source characterization likely helpful in regions with outdated, or lack of, bottom-up information to provide a swift insight of their emission sources. Overall, we find reasonable agreement between the satellite-based and the bottom-up emission categorization in 2010. However, the satellite perspective suggests an emission landscape less dominated by industrial areas, a larger role for transportation/urban areas and agricultural activities in East China, and more natural areas in the southeastern part of China in 2010.

We applied the same classification scheme in 2016 to evaluate whether the reduction in the NO_x_ and SO_2_ emissions is reflected by changes in our emission source classification. In 2016, from the OMI perspective, we find a 5% increase of nature-dominated sources, a 4% increase of agricultural lands, and a 9% decrease of urban (industry- and transportation-dominated) areas, relative to 2010. Approximately 65% of the emissions classified over East China has remained the same between 2010 and 2016. This suggests an overall decrease in heavy air pollution in East China in the span of 7 years with the most notable reduction of air pollution in the south (including the Pearl River Delta and the Yangtze River Delta). This reflects the extensive efforts by the Chinese government to improve the air quality in China, especially since 2013 when the National Air Pollution Prevention and Control Action Plan was enacted.

However, the southeastern provinces of Jiangxi and Zhejiang, and the southwestern provinces of Sichuan and Yunnan, and surroundings, show signs of urbanization. China has witnessed fast urbanization in past decades among which the population migration from rural to urban regions, in addition to the population growth. This resulted in stronger emissions from sources representing consumption and transportation which are strongly related to NO_2_ and PM_10_ pollution (rather than SO_2_) and are directly influenced by the population size. The “Go West” notion appears as a general movement of industrial and transportation activities from East to North and North-West of East China, favouring the expansion of city clusters and the creation of new air pollution hotspots. These changes are detected in this study, as 12% of previously flagged low emission areas have transitioned into areas with heavier pollution (mostly in the North and Northwest of East China), while 23% of previously flagged high emission areas have shifted to cleaner conditions (mostly in the East). Such changes also reflect the close relationship between population migration from/to urban areas and factors such as gender, age, educational background, and household income.

This study of relying only on satellite NO_x_ and SO_2_ observations, and their SO_2_:NO_x_ ratio, shows that it is possible to establish emission shifts from previously heavily polluted regions to cleaner areas, and emission trends due to increased industrialization. Overall, the effectiveness of the Chinese environmental control policies has been successful yet the air pollution in East China remains an important concern.

### Supplementary Information

Below is the link to the electronic supplementary material.

Supplementary file1 (DOCX 3.98 MB)

## Data Availability

The top-down NO_x_ and SO_2_ emissions are found on the globemission.eu portal; the top-down CO emissions are found on the https://emissions.aeronomie.be portal; the bottom-up NO_x_ and SO_2_ emissions (MIX inventory) are found on the http://www.meicmodel.org/dataset-mix portal; the agricultural NO_x_ emissions (REAS inventory) are found on the http://www.nies.go.jp/REAS/ portal; the land use data (GlobCover dataset) are found on the http://due.esrin.esa.int/page_globcover.php portal.

## Footnotes

1 The Supplemented MIX (SMIX) inventory contains (a) MIX SO_2_ and NO_x_ emissions from industry, power, transportation, and residence; (b) REAS soil NO_x_ emissions from agriculture (fertilizer application and livestock); (c) natural soil NO_x_ derived based on China-specific soil NO_x_ emissions studies and REAS agricultural soil NO_x_ emissions.
