# Supplementary material for: OMI-based emission source classification in East China and its spatial redistribution in view of pollution control measures
Source: Environ Monit Assess. 2024 Feb 29;196(3):323. doi: 10.1007/s10661-024-12421-8 (PMC10904434; doi:10.1007/s10661-024-12421-8)
Supplement: Supplementary file 1 — Supplementary file1 (DOCX 3.98 MB) [file 10661_2024_12421_MOESM1_ESM.docx]

*Supplement of*

OMI-based emission source classification in East China and its spatial redistribution in view of pollution control measures

Zara et al.

*Correspondence to*: folkert.boersma@wur.nl


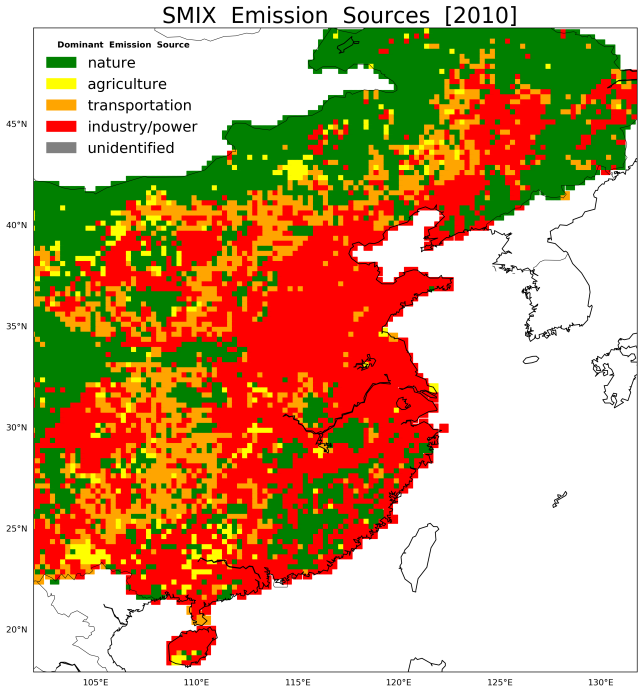

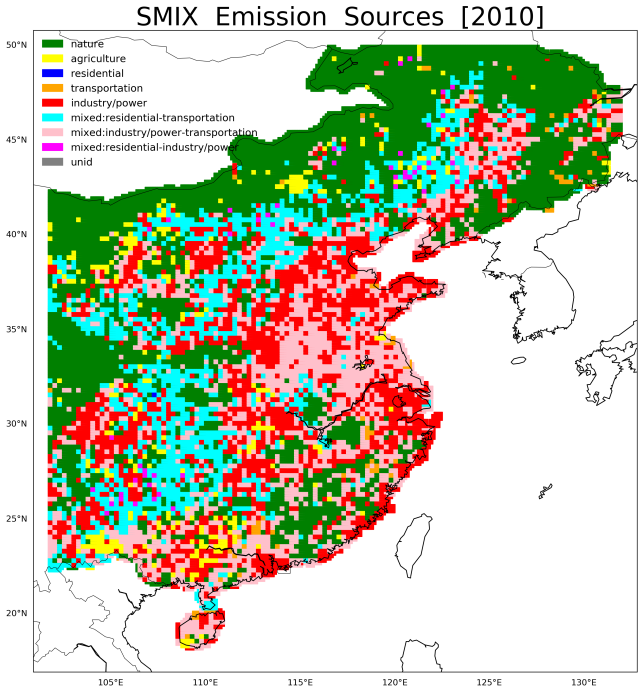


Figure S1 (left) Pre-classification of SMIX emission sources before the determination of the dominant emission source. The spatial resolution is 0.125°×0.125°, (right) Classification of SMIX emission sources and determination of the dominant emission source (Step 1 in Figure 3) for East China in 2010. The spatial resolution is 0.25°×0.25°.

Figure S1 (left panel) shows the SMIX emission categories before the selection of the dominant source is made. This classification still has mixed cells combining two emission categories (e.g. 16% ‘residence-transportation’, 21% ‘industry-transportation’, 0.6% ‘industry-residence’). After step 1 of the identification process discussed above and depicted in Figure 3, the dominant SMIX emission category is established resulting in 4 categories: industry/power, transportation, agriculture and nature (Figure S1; right panel). The number of occurrences (i.e. grid cells) where the highest NO_x_ and SO_2_ emissions are both originating from residential sources is extremely low (0.01%) therefore the residence category is merged with the transportation category since transportation is strongly associated with populated areas.

Figure S2 Cumulative frequencies of SMIX (top) SO_2_ (as S), (middle) NO_x_ (as N) and (bottom) SO_2_:NO_x_ (as S:N) of the cross-evaluated emission categories in ascending OMI SO_2_ and NO_x_ emission strength: nature - agriculture, agriculture - transportation, transportation – industry/power. The limit indicated as the meeting point of the two curves denotes the transitioning point between the two categories. The cumulative frequency of the category with the higher emission strength is shown reversed (i.e. from 100% to 0%).


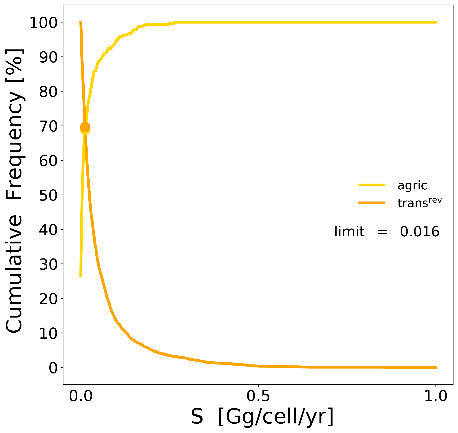

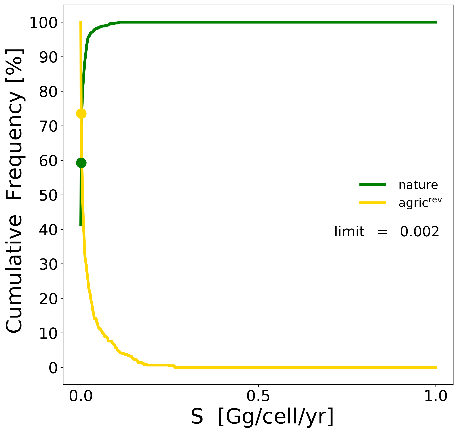

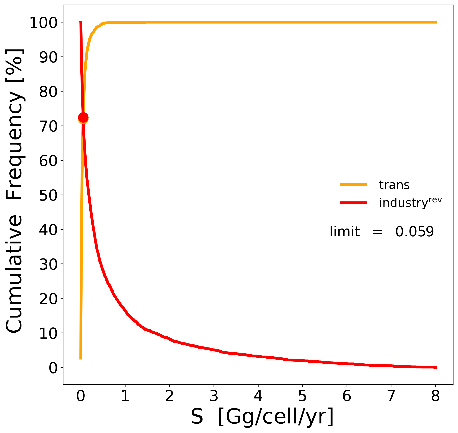

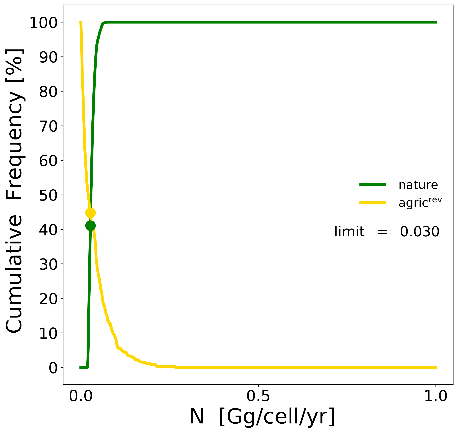

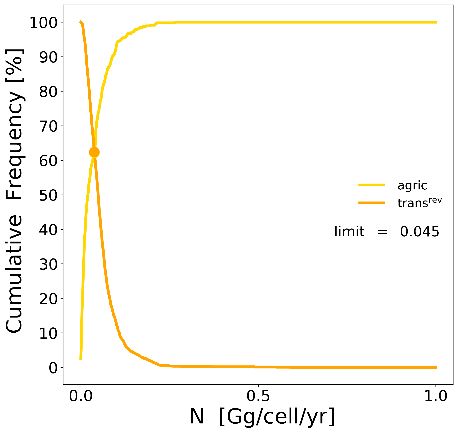

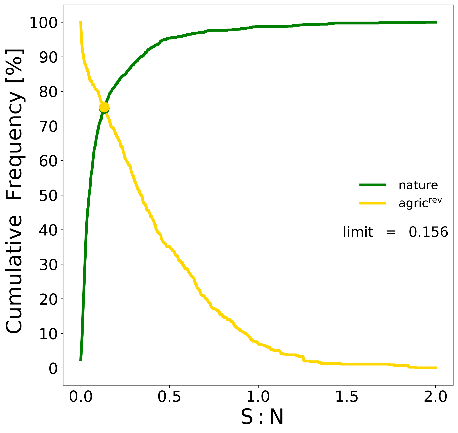

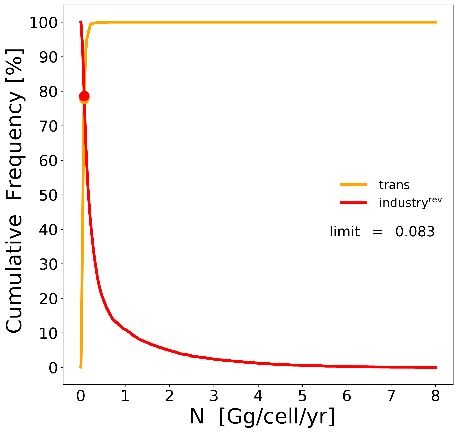

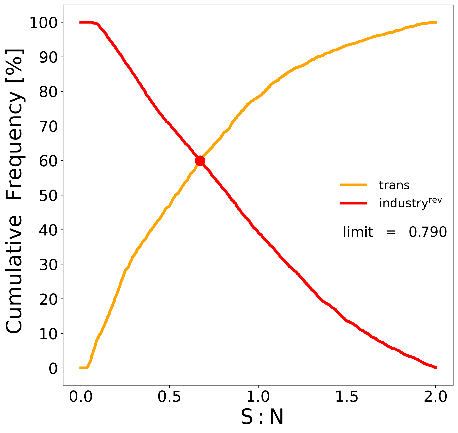

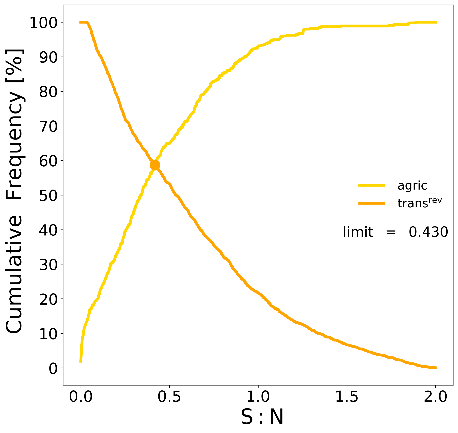


**Scheme that utilizes the limits detected between cross-evaluated emission categories and yield the dominant source type**

In Python 3.7.9 the following (*here* simplified) scheme was applied to all OMI grid cells to determine which emission category each cell belongs to.

For a grid cell of latitudinal position *lt* and longitudinal position *ln*:

if nox[lt, ln] == 0 and so2[lt, ln] == 0: emission_source[lt,ln] = unidentified

elif nox[lt, ln] < 0.030 or ratio[lt, ln] > 0.156: emission_source[lt,ln] = nature

elif nox[lt, ln] > 0.030 and nox[lt, ln] < 0.045: emission_ source[lt,ln] = agriculture

elif ratio[lt, ln] > 0.156 and ratio[lt, ln] < 0.430 : emission_source[lt,ln] = agriculture

elif nox[lt, ln] > 0.045 and nox[lt, ln] < 0.083: emission_source[lt,ln] = transportation

elif ratio[lt, ln] > 0.430 and ratio[lt, ln] < 0.790: emission_source[lt,ln] = transportation

elif nox[lt, ln] > 0.083 or ratio[lt, ln] > 0.790: emission_source[lt,ln] = industry

else:

sources[lt,ln] = ‘unidentified’

where *nox* denotes the NO_x_ emissions (expressed in Gg N/cell/yr), *so2* denotes the SO_2_ emissions (expressed in Gg S/cell/yr) and *ratio* denotes the SO_2_/NO_x_ ratio (expressed in S/N).

**Table S1** CO emissions maximum expressed as the 3^rd^ quantile plus 1.5× the inter-quantile range, the 90^th^ percentile, the 95^th^ percentile, and expressed in the nominal way (i.e. the maximum CO emission data point) for the winter months of January and December (when crop biomass burning in North China Plain is minimum) and the summer months of May, June, July (when crop fires in North China Plain are maximum - especially in June) in 2010. We choose the value of 0.5 Gg CO/cell/yr (i.e. extreme winter CO emission value) as the threshold for grid cells with June satellite NO_x_ emission estimate contaminated by NO_x_ emissions originated by biomass burning in North China plain in the same month.

|  | Maximum   (no outliers) = Q3 + 1.5*IQR | 90^th^   percentile | 95^th^  percentile | Maximum   (nominal) |
| --- | --- | --- | --- | --- |
| January | 0.000 | 0.000 | 0.004 | 0.433 |
| May | 0.016 | 0.050 | 0.101 | 2.266 |
| June | 0.028 | 0.120 | 0.360 | 4.737 |
| July | 0.007 | 0.024 | 0.055 | 1.492 |
| December | 0.000 | 0.000 | 0.004 | 0.400 |


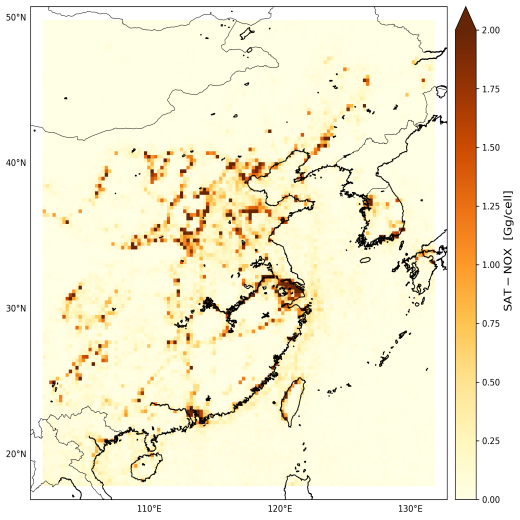


Before


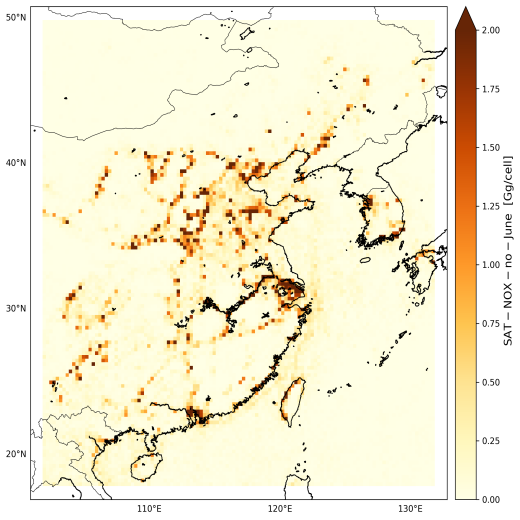


After


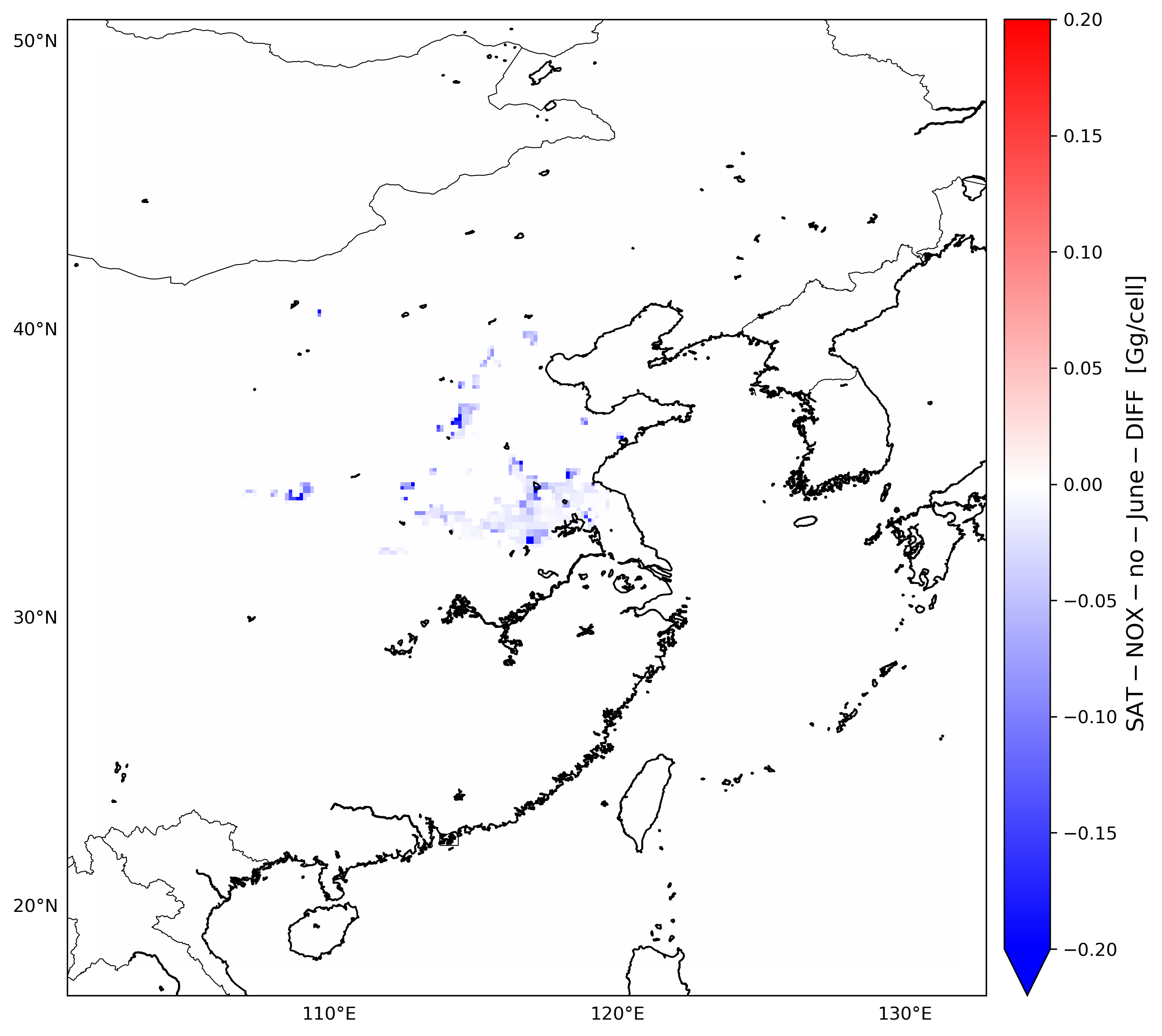


Difference

Figure S3 OMI-derived NO_x_ emissions before (left) and after (right) the exclusion of the month of June in the 2010-emission estimate for grid cells that yield CO emissions > 0.5 Gg CO/cell/yr. The difference is shown in the right panel depicting the enhanced crop fires that occur in North China Plain; 708 grid cells were found with June-CO emissions > 0.5 Gg/cell/yr. For these grid cells June is contaminated by the crop fire season and removed from their annual NO_x_ emission estimate. The spatial resolution is 0.125°×0.125°.

**Table S2**: GlobCover2009 v2.3 land use classification

| 1 | Post-flooding or irrigated cropland | 13 | Shrubland |
| --- | --- | --- | --- |
| 2 | Rainfed cropland | 14 | Herbaceous vegetation |
| 3 | Mosaic cropland (50-70%) / vegetation (20-50%) | 15 | Sparse (<15%) vegetation |
| 4 | Mosaic vegetation (50-70%) / cropland (20-50%) | 16 | Closed to open broadleaved forest regularly flooded |
| 5 | Broadleaved evergreen or semi-deciduous forest (>5m) | 17 | Closed broadleaved forest / shrubland permanently flooded |
| 6 | Closed broadleaved deciduous forest (>5m) | 18 | Grassland / woody vegetation regularly flooded |
| 7 | Open broadleaved deciduous forest / woodland (>5m) | 19 | Urban areas > 50% |
| 8 | Closed needleleaved evergreen forest (>5m) | 20 | Bare areas |
| 9 | Open needleleaved deciduous or evergeen forest (>5m) | 21 | Water bodies |
| 10 | Closed to open mixed broadleaved and needleleaved forest | 22 | Permanent snow and ice |
| 11 | Mosaic forest or shrubland (50-70%) / grassland (20-50%) | 23 | Inland water |
| 12 | Mosaic grassland (50-70%) / forest or shrubland (20-50%) |  |  |


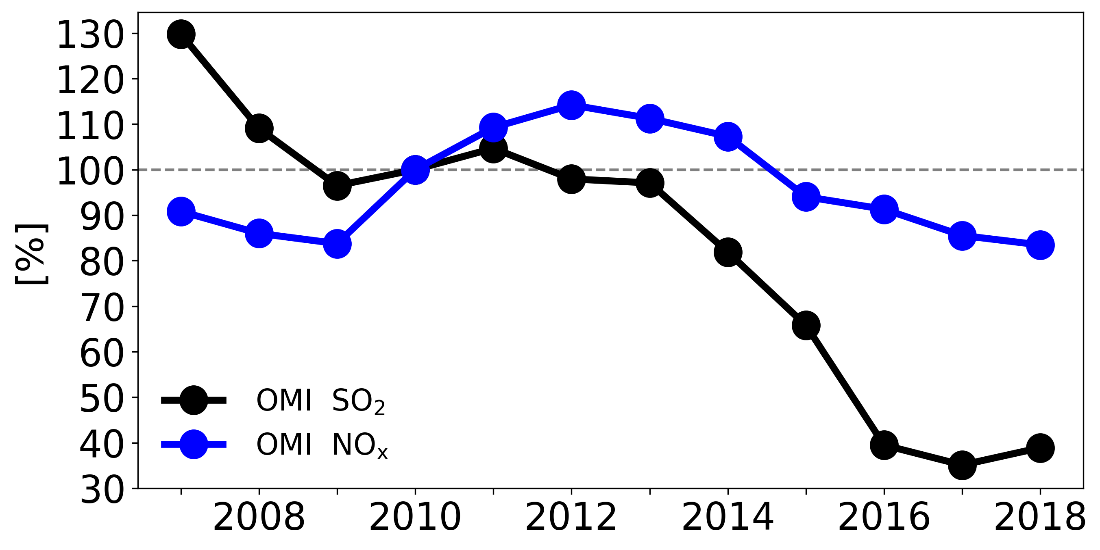


Figure S4 Time series of OMI-derived SO_2_ and NO_x_ emissions in East China for 2007-2018 normalized in 2010.
